# Single-port one-stage bilateral thoracoscopic sympathicotomy for severe hyperhidrosis: prospective analysis of a standardized approach

**DOI:** 10.1186/1749-8090-8-216

**Published:** 2013-11-26

**Authors:** Michiel Kuijpers, Theo J Klinkenberg, Wobbe Bouma, Mike J DeJongste, Massimo A Mariani

**Affiliations:** 1 Department of Cardiothoracic Surgery, University Medical Center Groningen, P.O. Box 30001, 9700 RB Groningen, The Netherlands; 2 Department of Cardiology, University Medical Center Groningen, Groningen, The Netherlands

**Keywords:** Single-port, Hyperhidrosis, Sympathectomy, Sympathicotomy, VATS

## Abstract

**Background:**

Primary palmar and/or axillary focal hyperhidrosis is a frequent disorder characterized by excessive sweating beyond physiological needs, often leading to a substantial impairment of quality of life. Over the years several minimally invasive surgical treatments have been described, however results vary, and due to a lack of uniform surgical approach, technique and nomenclature are often difficult to compare. In this prospective study we sought to evaluate the safety and effectiveness of our standardized technique of single-port, one-stage bilateral thoracoscopic sympathicotomy.

**Methods:**

On a prospective basis a hundred consecutive patients with severe or intolerable primary hyperhidrosis underwent one-stage bilateral single-port thoracoscopic sympathicotomy. Primary outcome was measured in pre- vs. post-operative Hyperhidrosis Disease Severity Scale scores. Location and extend of compensatory hyperhidrosis, and satisfaction with the procedure were registered.

**Results:**

A significant reduction in mean Hyperhidrosis Disease Severity Scale score (3.69 ± 0.47 preoperatively vs. 1.06 ± 0.34 postoperatively) (p < 0.001) was observed. In 97 (97%) out of the 100 enrolled patients a >80% reduction in sweat production was achieved. Compensatory hyperhidrosis was seen in 27 patients (27%). It was rated as mild by 21 patients (78%) and as moderate by 6 (22%) of these patients. No severe compensatory hyperhidrosis was reported. Major complications, such as intraoperative bleeding, infections, and Horner’s syndrome were not observed.

**Conclusions:**

Highly selective sympathicotomy at well-defined levels with a one-stage bilateral single-port transaxillary thoracoscopic approach is a save procedure, with excellent and reproducible immediate results in the treatment of primary palmar and/or axillary hyperhidrosis.

## Background

Primary hyperhidrosis (PHH) is characterized by excessive sweat secretion beyond the need for physiological thermoregulation without an underlying systemic condition. PHH tends to begin around the mid second to early third decade of life, affects men and woman equally and has a focal and often symmetric character [1]. It typically affects hands, feet and axillae, although combined palmar-axillary and craniofacial forms are relatively common as well.

Severe palmar and/or axillary focal PHH leads to feelings of shame and low self-esteem resulting in withdrawal from social activities, negatively affecting relational and professional development [2-4]. Although PHH is considered a benign disorder, it may have a deleterious impact on health related quality of life (HR-QoL). The reality that the latter has been underestimated until very recently is supported by the increasing reported prevalence in the literature, growing from 0.6% in 1977 to a recent 4.6% of the general population [1,5,6]. This observation substantiates that we might be looking at the tip of the iceberg called PHH. Non-surgical treatment modalities, like topical solutions, iontophoresis and botulinum toxin injections have proven to be temporarily successful in the treatment of PHH. However, non-surgical treatment modalities strain health care resources because of their temporary relief and demand a high level of patient compliance due to the need for repetitive administration. And while acceptable results have been achieved with local surgery that aims at the eccrine-gland level [7], these strategies are quite invasive and by nature only able to relieve axillary PHH.

Minimal invasive endoscopic thoracic sympathectomy (ETS) has become a well-established treatment modality and first-line treatment in palmar PHH. Large series have shown success rates of 71-100% following conventional ETS for all forms of PHH with improved quality of life and treatment satisfaction of 93-95% [8,9]. Recent experience is obtained with even less-invasive surgery and single-port access techniques. Since results, methods, nomenclature and used instruments vary widely, we sought to study the feasibility of our standardized, one-stage bilateral, single-port sympathicotomy.

## Methods

### Patients

In the present study we assessed a consecutive cohort of a hundred patients, suffering from severe or intolerable palmar and/or axillary PHH, who underwent a one-stage bilateral, single-port thoracoscopic sympathicotomy at our University institution. A diagnosis of primary focal hyperhidrosis was made according to the diagnostic criteria described by Hornberger et al. [10]. These state that 'focal, visible, excessive sweating for at least 6 months without secondary cause’ has to be present, combined with at least two of the following characteristics: bilateral and relative symmetric sweating, frequency of at least one episode per week, impairment of daily activities, age at onset <25 years, positive family history and finally cessation of sweating during sleep. The HDSS questionnaire (See Table 1) was used for adequate selection of patients preoperatively and for quantification of the effect of sympathicotomy, postoperatively. A 1-point improvement in HDSS score is associated with a 50% reduction in sweat production; a 2-point improvement constitutes an 80% reduction [11]. A pre-operative HDSS score of ≥3, classifying hyperhidrosis as severe or intolerable, was mandatory to qualify for surgery. Informed consent was obtained from all participants.

**Table 1:** **Hyperhidrosis disease severity scale **[11]

**'How would you rate the severity of your sweating?’**	**PHH severity**
*1. 'My sweating is never noticeable and never interferes with my daily activities’.*	*Mild*
*2. 'My sweating is tolerable but sometimes interferes with my daily activities’.*	*Moderate*
*3. 'My sweating is barely tolerable and frequently interferes with my daily activities’.*	*Severe*
*4. 'My sweating is intolerable and always interferes with my daily activities’.*	*Intolerable*

Assessments for quantifying the social impact and dermatologic manifestations of severe or intolerable PHH (HDSS score of ≥3), was performed with the Skindex-29 [12], a well-validated psychometric dermatologic HR-QoL questionnaire. The value of the Skindex-29 has been thoroughly evaluated, with multiple studies into cut-offs and the interpretation of its scores [13]. The Skindex-29 consists of 30 items related to the impact of PHH on every-day life on a five-point response scale. Twenty-nine out of the 30 items are assigned to three scales, separating scores into impact on emotions, functioning and symptoms. Main parameters affecting post-operative satisfaction with the therapy were recorded, such as incidence and location of Compensatory Hyperhidrosis (CHH). Moreover, overall patient satisfaction of the performed sympathicotomy was measured using a five-point satisfaction scale. The following grading was used: very satisfied, satisfied, neither satisfied nor dissatisfied, somewhat dissatisfied, or very dissatisfied.

Chest X-ray and routine blood and urine testing were performed prior to surgery to exclude gross pulmonary or pleural abnormalities and infections. The HDSS was repeated at 2-weeks follow-up to quantify obtained results. Preoperative patient data are presented in Table 2.

**Table 2:** Preoperative patient data (n = 100)

**Variable**^**(a)**^	**Value**
Age (y)	31.1 ± 10.0
Gender	
Male	49 (49)
Female	51 (51)
Intervention	
Primary thoracic intervention	99 (99)
Re-thoracic intervention after congenital cardiac surgery through a left thoracotomy	1 (1)
Form of PHH	
Isolated Palmar	25 (25)
Isolated Axillary	37 (37)
Combined Palmar and Axillary	38 (38)
HDSS Classification	
Class 1 (mild)	0 (0)
Class 2 (moderate)	0 (0)
Class 3 (severe)	31 (31)
Class 4 (intolerable)	69 (69)

^(a)^Data are presented as mean ± standard deviation or number (%).

HDSS = Hyperhidrosis Disease Severity Scale.

Patients were prescribed analgesic medication (Paracetamol 4 dd 1000 mg / Tramadol 3 dd 50 mg / Diclofenac 3 dd 50 mg) starting 2 hours after the procedure, and continuing for one week post-operatively.

### Surgical technique

In 2011 we reported on bilateral single-port thoracoscopic sympathicotomy with the VasoView® device [14]. In this preliminary study, the patient was positioned on the left side with the arm flexed over the head to expose the axilla. After a right-sided sympathicotomy the patient was repositioned on the right side and prepped and draped again to undergo a left-sided sympathicotomy. Improving operative time and patient safety, we adopted the beach-chair or semi-fowler’s position, originally developed in the 1980s for orthopedic shoulder arthroscopy procedures. Patients were seated at a 45° angle above the horizontal plane with appropriate padding and with their head secured in a headrest. The bilateral axillary exposure achieved in this way allows for a one-stage bilateral procedure while the obtained angle helps folding the collapsing lung not only in dorsal, but also in caudal direction, facilitating a complete view of the surgical field. This can be of help in sympathicotomy of R5 where the downwards-folded lung allows for easier access to the fifth rib.

General anesthesia was administered and single lung ventilation was obtained using a double lumen endotracheal tube (53 patients), or single lumen endotracheal tube (3 patients) with unilateral blocker (EZ-blocker Inc, Delft, The Netherlands). In time we switched to a single lumen endotracheal tube, performing the sympathicotomy during short pauses in ventilation in the last 46 patients of the cohort. Following local infiltration with bupivacaine, a 6 to 7 mm incision was made in the right anterior axillary line. After deflation of the lung, a 5 mm inner diameter trocar was inserted through the third intercostal space just posterior to the major pectoral muscle. CO_2_ insufflation was initiated when collapse of the lung proved to be insufficient. A 5 mm scope (Karl Storz, Tuttlingen, Germany) and cautery hook were then introduced. The first and second rib as well as the sympathetic chain running along the neck of the ribs were identified. The part of the sympathetic chain overlying the rib was transected by diathermy on a high costal level, sparing the sympathetic ganglia. A R3 sympathicotomy was performed for isolated palmar PHH and a R3-R5 sympathicotomy for axillary or combined palmar/axillary PHH. In all cases the transection was extended 2 cm laterally over the rib to transect accessory nerve fibers. Active search for the presence of Kuntz’s nerve was performed and transected when present. The surgical procedure was completed by inspection of the collapsed lung for parenchymal damage and insertion of an 8 French thoracic drain through the same access port. Re-insufflation and recruitment of the collapsed lung was done under direct vision. The thoracic drain was removed under positive end expiratory pressure. A thoroughly placed subcutaneous string suture ensured airtight incision sealing. The skin was closed with an intracutaneous suture. The same procedure was then repeated on the left side. Operation time, level of sympathicotomy and duration of hospital stay were recorded and presented in Table 3.

**Table 3:** Surgical data (n = 100)

**Variable**^**(a)**^	**Value**
Operative Time (min)	47 ± 18
Duration of Hospital stay (days)	1.15 ± 0.67
Day-care	45 (45)
Non day-care	55 (55)
Level of sympathicotomy	
R3	25 (25)
R3-R5	75 (75)

^(a)^Data are presented as mean ± standard deviation or number (%).

### Statistical analysis

Continuous variables were expressed as mean ± standard deviation. Categorical variables were expressed as percentages. Pre- and postoperative HDSS score comparisons were performed using the paired samples *t*-test. Data were displayed in a box-and-whisker plot. All calculations were performed using a commercially available statistical package (IBM SPSS Statistics for Windows, Version 20.0. Armonk, NY: IBM Corp). Statistically significant differences were established at *p* < 0.05.

## Results

### Operation time and hospital stay

Mean operative time was 47 minutes for a bilateral sympathicotomy in beach-chair position. A significant reduction in mean operative time was achieved compared to the staged procedure [14] (74 ± 12 vs. 47 ± 18, p <0.001). The average hospital stay was 1.15 (1–6) days, allowing for 45 (45.0%) day-care based procedures.

### Complications and postoperative pain

Complications, such as intraoperative bleeding, infections, and Horner’s syndrome were not observed. Conversion to a thoracotomy or placement of extra ports was not required in any patient. A post-operative pneumothorax was seen in four patients (4%), requiring drainage using a pleural catheter in three patients. All patients fully recovered and were discharged the day after, following chest X-ray examination and drain removal. Postoperative pain requiring analgesics for longer than one week (standard prescription) was recorded in thirteen patients (13%) with only three patients (3%) requiring more than paracetamol at two-week follow-up (17.3 ± 6.1 days).

### Pre- and postoperative HDSS scores

Pre-operative thirty-one patients (31%) reported a HDSS score of 3 (severe symptoms) and sixty-nine patients (69%) reported a HDSS score of 4 (intolerable symptoms). Post-operative at two-week follow-up ninety-six patients (96%) were in HDSS class 1 (no or mild symptoms), and three patients (3%) were in HDSS class 2 (moderate symptoms). One patient (1%) remained in HDSS class 4, due to only right-sided success. Mean HDSS score pre-operative was 3.69 ± 0.47 and mean post-operative HDSS 1.06 ± 0.34 (*P* < 0.001). Ninety-seven patients (97%) reported post-operative a ≥2-point reduction in HDSS associated with an 80% reduction in sweat production. Two patients (2%) had a 1-point reduction in HDSS associated with a 50% reduction in sweat production. In one case (1%) the HDSS score was not affected by the intervention.

### Compensatory hyperhidrosis and patient satisfaction

After two weeks of follow-up (17.1 ± 6.3 days) CHH was seen in twenty-seven patients (27%), and was not dependent on the level of sympathicotomy. When asked to rate the severity of their CHH it was rated as mild by twenty-one patients (78%) and as moderate by six patients (22%). No severe or intolerable CHH were reported. Affected body areas were abdominal in two patients (7.4%), feet in three patients (11.1%), legs/groin in five patients (18.5%), and lower back in seventeen patients (63.0%). Distribution of CHH severity was not related to affected body areas. It is of interest to note that 25 of the 27 patients (93%) suffering from CHH scored at follow-up HDSS class 1 'my sweating is never noticeable and never interferes with my daily activities’. Two patients suffering from CHH reported a HDSS class 2 'my sweating is tolerable but sometimes interferes with my daily activities’. High satisfaction rates were observed; eighty-nine patients (89%) were very satisfied, ten patients (10%) were satisfied with the post-operative result. One patient (1%) was unsatisfied with the procedure because of unilateral success. No patient regretted having undergone the procedure.

### Skindex-29 data

Preoperative Skindex-29 data are shown in Table 4. The Skindex-29 dataset was incomplete for three patients and these scores were excluded. These data clearly shows the negative impact on quality of life of patients with severe and intolerable PHH; sixty-five patients (67.0%) have a severe overall score and thirteen patients (13.4%) have a moderate overall score. Average scores on seven individual questions particularly stand out providing an illustrative picture on emotional stress and impact on social functioning (See Table 5).

**Table 4:** Preoperative skindex-29 scores (n = 97)

	**Skindex-29 domains**^**(a)**^			
	**Symptoms**	**Emotions**	**Functioning**	**Overall**
	19.5 ± 17.1	57.7 ± 18.5	54.2 ± 18.2	46.8 ± 14.7
**Severity**^(b)^				
Little (< Mild)	84 (86.6)	4 (4.1)	5 (5.2)	8 (8.2)
Mild	2 (2.1)	9 (9.3)	8 (8.2)	11 (11.3)
Moderate	8 (8.2)	7 (7.2)	2 (2.1)	13 (13.4)
Severe	3 (3.1)	77 (79.4)	82 (84.5)	65 (67.0)

^(a)^Domain and Overall scores are expressed on a 100-point scale; higher scores indicate lower quality of life levels, presented as mean ± standard deviation.

^(b)^Anchor-based cut-off scores, Prinsen et al. [13] presented as number (%).

**Table 5:** Preoperative skindex-29 top-7 scores (n = 97)

**Question**	**Value**^**(a)**^
1. 'I am annoyed by my skin condition’.	84.8
2. 'I am embarrassed by my skin condition’.	84.5
3. 'I am ashamed of my skin condition’.	84.5
4. 'My skin condition affects my social life’.	83.3
5. 'My skin condition makes it hard to work or do hobbies’.	78.9
6. 'My skin condition affects my interaction with others’.	76.6
7. 'I am frustrated by my skin condition’.	72.9

^(a)^Average scores, expressed on a 100-point scale. Higher scores indicate lower level of quality of life levels.

## Discussion

Various HR-QoL instruments have been described for impact measurement and intervention-effect quantification in PHH, including the Short Form 36 (SF-36), and a proposed disease specific questionnaire [9]. The sensitivity of a general HR-QoL instrument like the SF-36 is however limited by good general health status of this young, otherwise healthy population. Since hyperhidrosis specific QoL questionnaires often lack repeated validation and are not frequently used in the context of surgical intervention thus hindering comparison of results, we opted for the Skindex-29, a well-validated psychometric dermatologic questionnaire.

In the present study, in 67.0% of patients the overall Skindex-29 rating showed a severe impact on their quality of life. Interestingly, severe hyperhidrosis has a different impact on the three different HR-QoL domains measured. The severe impact on the emotions- and functioning-domains in well over three quarters of patients (79.4% respectively 84.5%) demonstrates the great emotional stress and seriously impaired social and professional functioning induced by severe PHH. In the symptoms-domain however, only 3.1% of patients rated a severe impact. This can be explained by the fact that hyperhidrosis produces little physical symptoms included in Skindex-29 like itching, bleeding and pain, and because of the fact that patients try to hide PHH reducing the impact of symptoms on HR-QoL. This strengthens the notion that PHH is a highly underestimated medical and social problem. In fact, several studies have shown the impact of hyperhidrosis on quality of life to be highly comparable to severe psoriasis, multiple sclerosis, vitiligo, rheumatoid arthritis and even end-stage renal failure [7,15-17].

The HDSS proofs a valuable asset for patient selection and quantification of the perceived severity of PHH both pre- and postoperatively. Although not specifically designed for CHH impact detection, the post-operative HDSS seems to capture the true social impact of the sympathicotomy. Since CHH, when present, will influence the postoperative HDSS score, it provides a very easy to obtain, yet comprehensive outcome assessment.

This study reports the data of the further development of the previously described single-port operative technique [14] with the patient now positioned in beach chair position. Although not yet standard practice in most research groups we propose it should be since this position avoids possible complications associated with repositioning intubated and anaesthetized patients. Moreover it provides an easy access for one-stage bilateral sympathicotomy and reduces procedure time.

Despite proposed uniform terminology and nomenclature in describing sympathetic chain surgery, exact definitions regarding surgical access and level and type of sympathetic transection still vary greatly [18-22], while also different approaches for the different levels of intervention have been described [20]. Nomenclature of interventions like clipping, true sympathectomy (resection or ablation of a portion of the sympathetic chain including the ganglia) and sympathicotomy on different levels are variably used, leading to confusing results and hindering interpretation and comparison of results reported by different authors. In this study, we used the set of common definitions and standardized nomenclature as recently proposed by the Society of Thoracic Surgeons through Cerfolio et al. [23]. We wholeheartedly support these uniform terms, and their international future use. We believe they should be used by all groups who are working in the field of 'surgical treatment of hyperhidrosis’.

It is known that a single-port thoracoscopic approach causes less postoperative pain and a shorter operation and recovery time [18]. Better functional and cosmetic results of a truly minimally invasive single-port access compared with more conventional, but still frequently used bi- or tri-portal approaches render these multi-port strategies obsolete. In our study 45 patients (45%) were treated on outpatient basis. This percentage does not reflect medical implications since all patients were offered the possibility to stay the night following the procedure. This was done to reduce travelling risks because of the large referral area and to increase patient comfort. Three patients had to stay overnight for medical reasons (pleural drainage).

In recent literature high satisfaction rates (96.6% vs. 89.6%) combined with a significant lower incidence of severe CHH (3% vs. 10%) are obtained by performing only a R3 sympathicotomy for the treatment of PHH, and a R3-R5 sympathicotomy for combined palmar and axillary PHH [19]. According to this evidence, we performed a R3 sympathicotomy for isolated palmar PHH and a R3-R5 sympathicotomy for axillary or combined palmar/axillary PHH. By cauterizing the sympathetic chain at the top of R3, or top of R3-R5 while leaving the R2 level and all sympathetic ganglia intact, we performed a true sympathicotomy in this study. Very high rates of satisfaction (99%) and success (97% of patients had an 80% reduction in sweat production) were observed. Twenty-seven percent of patients reported CHH with an acceptable incidence of mild CHH (21%) and moderate CHH (6%) CHH. Severe CHH did not occur.

Opposite effects of sympathicolysis level on CHH have also been reported, with a low incidence of CHH after isolated R2 sympathectomy (13%) compared to R2-R4 sympathectomy (34%) [20]. However, patients who had division of the sympathetic chain at multiple levels, showed a significantly higher incidence of CHH. The high success-rates obtained in our study, with a relative low incidence of CHH, strengthens the theory that including the R2 level is not required for adequate PHH relief in any form of isolated palmar or combined palmar/axillary PHH, and is thus better avoided since it heightens the risk for Horner’s Syndrome [21]. It’s also known that a more extensive sympathetic denervation is correlated to a significant higher disturbance in bronchomotor tone and cardiac function, albeit on a sub-clinical level [24]. Perhaps the overlapping conclusion should be that less is more, meaning that lower CHH levels can be achieved by less damage to the sympathetic chain with a more selective sympathicolysis. While in axillary hyperhidrosis a R3-R5 sympathicotomy is adequate, we feel that an isolated R3 sympathicotomy suffices in the treatment of palmar hyperhidrosis, possibly offering the best compromise between success in treating PHH and the risk of CHH, by averting intervention at multiple levels.

## Conclusions

Careful patient-selection using uniform, evidence based diagnostic criteria including disease-severity, a standardized operative procedure, unambiguous anatomical nomenclature, and adequate outcome assessment are key issues. Performing a highly selective sympathicotomy at well-defined levels using a single-port one-stage bilateral trans-axillary thoracoscopic approach, we combined a safe and minimally invasive surgical technique with excellent and reproducible immediate results in the treatment of primary palmar and/or axillary hyperhidrosis, rendering the practice of multi-port interventions in these patients superfluous. The HDSS was found an excellent measure with regard to patient selection, satisfaction and improvement in quality of life. Regardless of the excellent short-term results achieved, long-term follow-up studies including repeated HDSS and Skindex-29 questionnaires are needed to determine the life-long effects of thoracic sympathicotomy and its impact on everyday life for patients suffering from invalidating PHH.

## Abbreviations

CHH: Compensatory Hyperhidrosis; ETS: Endoscopic Thoracic Sympathectomy; HDSS: Hyperhidrosis Disease Severity Scale; HR-QoL: Health Related Quality of Life; PHH: Primary Hyperhidrosis; R2-5: Rib level 2–5.

## Competing interests

The authors declared that they have no competing interest.

## Authors’ contributions

MK and TK performed the operations and acquired the data. MK participated in the study design, performed the statistical analysis and interpretation of the data and drafted the manuscript. WB, MJ, TK and MM conceived of the study, participated in its design and coordination and helped to draft the manuscript. All authors read and approved the final manuscript.
